# Seed Quantity or Quality?—Reproductive Responses of Females of Two Dioecious Woody Species to Long-Term Fertilisation

**DOI:** 10.3390/ijms23063187

**Published:** 2022-03-16

**Authors:** Emilia Pers-Kamczyc, Ewa Mąderek, Jacek Kamczyc

**Affiliations:** 1 Institute of Dendrology, Polish Academy of Sciences, Parkowa 5, 62-035 Kórnik, Poland; maderek@man.poznan.pl; 2 Faculty of Forestry and Wood Technology, Poznan University of Life Sciences, Wojska Polskiego 71C, 60-625 Poznań, Poland; jacek.kamczyc@up.poznan.pl

**Keywords:** seed mass, seeds metabolome, seed size, English yew, common juniper, nutritional availability, nitrogen deposition, resource allocation

## Abstract

Although seed quality and quantity, as well as reproductive performance are important life history stages of plants, little is known about the reproductive responses of trees to environmental changes such as increased anthropogenic deposition of nitrogen (N) and phosphorus (P). Dioecious plants are good models with which to test the environmental impact on female or male reproductive responses individually. We analysed effects of different long-term nutritional availability on the reproductive performance of two dioecious species (*Taxus baccata* L. and *Juniperus communis* L.) characterised by different life histories. By using pot experiments with vegetatively propagated plants grown in different fertilisation conditions, we observed an increase in plant growth and strobili production but a decrease in seed efficiency. Seeds produced by fertilised plants had greater seed mass. Fertiliser addition did not change C or N content nor the C/N ratio of *T. baccata* seeds, but increased N content and the N/P ratio; however, it did lower the C/N ratio in *J. communis*. Fertilisation did not change the metabolite profile in *T. baccata* but 18 metabolites were changed in *J. communis*. The study revealed new links between species life history, environmental changes, and reproduction. The findings imply that future environmental conditions may alter both seed productivity, and quality, as well as plant reproductive behaviour.

## 1. Introduction

Seed recruitment is a primary factor determining the long-term dynamics of plant population in a changing environment; however, seed quality is also an important factor. The term “seed quality” describes the overall value of a seed lot based on many features, and is determined by genetic background and the environmental conditions of the mother plant during seed development [1]. The maternal plant allocates available resources to growth, defence, or reproduction [2], and, therefore, the resources available in the maternal environment can have a great impact not only on the quantity but mainly on the quality of seeds produced. The quality of the seeds is usually described by their weight and size, storability, vigour, germinability, concentration and nitrogen (N) content, as well as the development of the embryo.

Seed size is considered to be an important evolutionary trait that affects the reproductive success of many plant species, and variation in seed size is an important area of plant ecology [3]. Seed size can directly affect the processes of germination and seedling recruitment, and further impact plant performance under different environmental conditions. Smaller seeds generally germinate faster; however, although larger seeds germinate more slowly, their germination success is often higher than that of small seeds [4,5]. The weight and size of seeds are directly related to nutritional reserves; thus larger seeds can accumulate more resources available for seedling growth [6]. Moreover, seed N concentration (percentage N per unit seed mass) and N content (total N mass per seed) have been singled out as key proxies for seed quality, and a higher N content was positively related to the fitness, establishment, and survival of seedlings [7,8].

Reproductive output is also altered by climate and environmental changes (nutritional availability) and many studies have shown that temperature and rainfall can influence reproductive performance [9]. Many studies have shown that the environment has changed greatly due to the deposition of anthropogenic N, phosphorus (P), and other minerals in the soil and forest stands [10]. N deposition has increased since the 1900s, having already doubled since then, and is projected to double again by 2050 [11]. Although N is an essential plant nutrient and is described as a limiting factor, many terrestrial ecosystems have adapted to conditions of low N and P availability. However, plant ecosystems, including forests, are currently subjected to global changes, which have greatly modified the biogeochemical cycles of carbon (C), N, and P [11,12,13,14]. Airborne N input differs between ecosystems and it is estimated to be between 8–9 kg/ha/year [12]. These rapid global changes can pose a significant threat to plant species; thus, elevated N as well as P deposition negatively affect plant biodiversity and ecosystem functioning [12]. However, N supplies in forest stands also have a positive impact; thus, N deposition has led to an increase of aboveground biomass of forest trees [15], including rapid foliar growth [16] and seed production [17]. N is an important nutrient stored in seeds, but calcium (Ca), potassium (K), and magnesium (Mg) are also sequestered within mature seeds and can fulfil regulatory roles (e.g., osmoregulation, cell extension, and cell wall stabilisation) [18].

Although the environmental maternal effect on seed weight is important [19], there is a lack of knowledge about the environmental impact of different long-term nutritional availability on female reproductive potential and seed production; thus, available research shows contradicting data on the impact of N availability on female reproductive traits (mainly seed production [7,20,21]). Even though N-fertilisation increases seed production, it can also decrease seedling emergence and survival [20,21]. Moreover, negative impacts of high nutritional resource availability on male reproductive features are reported; thus, male individuals grown in an environment of rich fertiliser availability produced a large quantity, but lower quality, of pollen grains [22,23]. Unfortunately, female reproductive features have received less attention in the plant sciences than vegetative ones, except for seed size and weight [3].

Resource allocation in seeds is described as the economy of seeds, and is important for different stages of regeneration and needs to be considered in the larger context of recruitment processes [24]. The seed size–number trade-off is well understood as a major dimension of trait variation; however, other functional relationships between vegetative and seed traits are needed to integrate seed traits into their function, such as dispersal, persistence, germination timing, and seedling establishment [3]. Dioecious species are good models for studying environmental impacts on reproductive features. Males produce pollen grains and females produce seeds, and each process can be analysed without the influence of the other.

Many dioecious species are characterised by the presence of sexual dimorphism, which leads to spatial segregation between the sexes [25]. Moreover, they are particularly vulnerable to the effects of climate and environmental changes [26,27]. Most prior research analysed the impacts of environmental changes concerning dioecious plant growth, biomass allocation, and seedling survival, but the long-term impact on the reproductive success of plants has rarely been studied [28,29,30,31,32]. Thus, for many woody species, there is still a lack of a molecular sex marker, and the plant sex can be described only by reproductive structures generated on the shoots [33,34]. The only way to study the environmental impacts for female or male plants is, therefore, to use individuals produced by vegetative reproduction (rooted shoots) grown under similar conditions. This allows us to minimise the impacts of different genotypes on the observed plant traits, and to more precisely describe and test the effect of an experimental factor.

*Taxus baccata* L. and *Juniperus communis* L. are examples of dioecious plants characterised by different life-history strategies. *T. baccata* prefers rich habitats [35], while *J. communis* is a pioneering species found in sandy soil [36]. Both species are wind-pollinated, dioecious species, with male and female strobili growing on separate plants [35,36], and they have different phenological patterns. *T. baccata* plants produce seeds within a one-year cycle with the base of the strobila developing in the autumn of the previous year, whereas in *J. communis* the seed-producing cycle lasts up to three years [35,37]. These species are characterised by gender-related ecological differences between the sexes within species [38,39,40,41,42], and population decline [37,43,44]. Thus, female plants of both species allocate resources for growth, defence, and seeds, but not pollen production, and can be great models to test the impact of long-term different nutritional availability on the female reproductive responses.

Our research sought to determine the impacts of the availability of environmental nutrition on the reproductive success of female plants of two dioecious species (*Taxus baccata* L. and *Juniperus communis* L.) presenting different life histories and distinct phenological patterns when grown in long-term pot experimental conditions under two different nutritional regimes. For our study, we used rooted plants of both species, and plants produced from the same maternal origin were grown under two conditions—fertilised and non-fertilised. Therefore, the environmental effects on the reproductive outcomes were analysed from a more integrated perspective, in which we analysed the time required for plants to mature and to produce mature seeds, as well as many other quantitative and qualitative aspects of seed production. Within this study, we investigated the impacts of nutritional availability (fertilised (high N and P) vs. non-fertilised (low N and P)) on: (1) timing of plant maturity, (2) efficiency of seed production, (3) seed quality as described by seed weight, embryo development, and morphometric features, (4) timing of aril/cone maturity, and (5) content of macronutrients and GC-MS metabolite profile.

Taking all of the above into account, we hypothesised that higher nutrition availability will increase the productivity of female plants. We assumed that in a richer environment, plants will grow bigger and will be characterised by a higher productivity of female reproductive structures and seeds. Moreover, plants from fertilised conditions will be characterised by higher productivity, but the same quality of seeds’, compared to non-fertilised counterparts. In addition, fertilised plants will reach sexual maturity earlier than non-fertilised plants, but the time required for developing seeds to mature will be similar. Both species will exhibit a similar pattern of reproductive response.

## 2. Results

### 2.1. Plant Features

#### 2.1.1. Sexual Maturity of Plants

**Figure 1:**
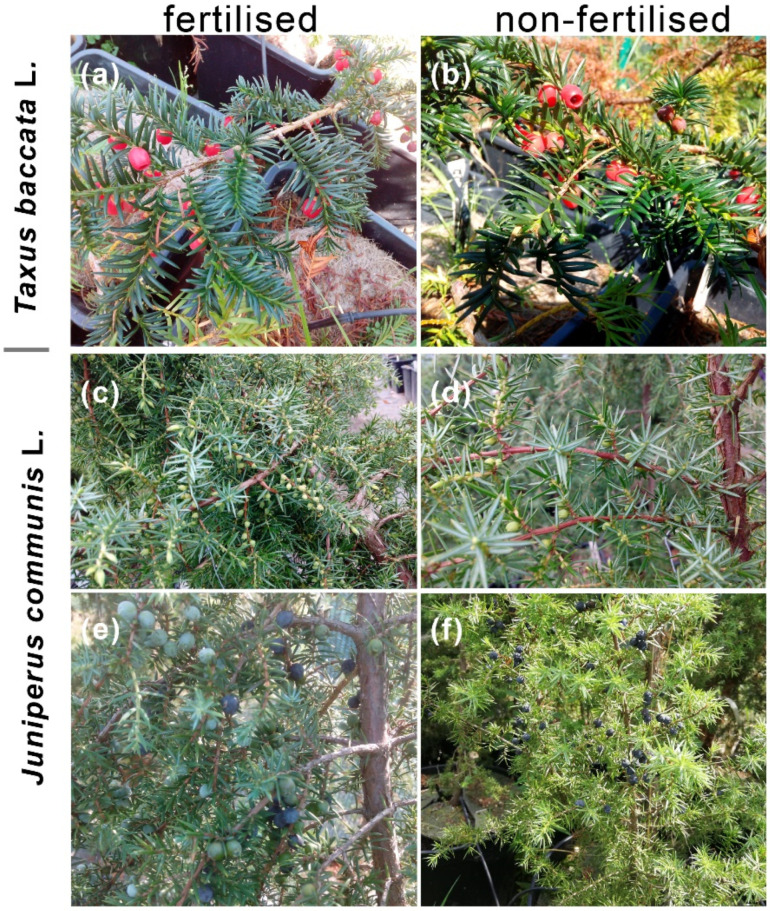
Features of *Taxus baccata* L. (**a**,**b**) and *Juniperus communis* L. (**c–f**) plants grown in fertilised (**a**,**c**,**e**) and non-fertilised conditions (**b**,**d**,**f**) within the observation period.

For *Taxus baccata,* none of the female plants produced ovules in 2013, and more fertilised than non-fertilised plants produced ovules in 2014 (24%, 6/25, from one to five ovules vs. 20%, 5/25, from one to four ovules) and 2015 (92%, 23/25, from two to 93 ovules vs. 76%, 19/25, from one to 17 ovules), whereas all plants from both treatment groups produced ovules in 2016. The number of ovules produced by fertilised plants in 2016 ranged from 28 to 657, whereas non-fertilised plants produced nine to 89 ovules (Figure 1a,b).

For *Juniperus communis*, none of the female plants produced flowers in 2013, more fertilised than non-fertilised plants produced female flowers in 2014 (23.08%, 6/26 vs. 7.69%, 2/26), 2015 (80.77%, 21/26 vs. 46.15%, 12/26) and 2016 (100% 26/26 vs. 84.61% 22/26), whereas all plants from both treatment groups produced female flowers in 2017 and 2018 (Figure 1c–f).

#### 2.1.2. Plants Morphology

For *T. baccata*, in 2016, fertilised plants were taller (F: 74.95 ± 3.26 cm vs. NF: 58.19 ± 3.07, *p =* 0.001) and produced similar numbers of shoots (F: 40.85 ± 3.38 vs. NF: 32.50 ± 2.69, *p =* 0.102*)* but shoots were longer (F: 9.36 ± 0.48 cm vs. NF: 6.60 ± 0.45 cm, *p <* 0.001) when compared with non-fertilised plants. Fertiliser availability had also affected main shoot diameter: fertilised plants were 11.93 ± 0.45 mm whereas non-fertilised were 9.12 ± 042 mm (*p <* 0.001).

For *J. communis*, in 2017, matured fertilised plants were taller (114.71 ± 2.92 cm vs. 99.73 ± 2.92, *p <* 0.001); however, in 2018, there was no statistically significant difference in height between fertilised and non-fertilised plants (F: 130.10 ± 4.13 cm and NF: 122.96 ± 4.22, *p =* 0.238), but fertilised plants had larger diameter (F: 24.39 ± 0.79 mm vs. NF: 18.50 ± 0.79, *p <* 0.001) and had longer shoots (located in the middle of the main stem: F: 21.23 ± 1.28 vs. NF: 12.78 ± 1.28, *p <* 0.001).

### 2.2. Quantitative Features of Female Plant Reproductive Response

#### 2.2.1. Number of Generative Structures

In *Taxus baccata*, in total, fertilised plants produced 4879 strobili in March 2016, of which 1843 were still present as developing ovules in June, resulting in a collection of 656 arils, whereas non-fertilised plants produced 1449 strobili, 868 ovules, and 517 arils. The mean number of strobili (F: 203.29 ± 20.35 vs. NF: 53.67 ± 19.19, *p <* 0.001), the mean number of ovules (F: 76.79 ± 11.17 vs. NF: 32.15 ± 10.54, *p =* 0.005), but not the mean number of arils (F: 27.33 ± 4.14 vs. NF: 19.15 ± 3.90, *p =* 0.16) produced per plant were higher in fertilised plants when compared with non-fertilised plants. Furthermore, fertilised plants produced more female strobili (F: 30.50 ± 3.27 vs. NF: 9.84 ± 3.08, *p <* 0.001) but similar mean number of arils (F: 3.79 ± 0.57 vs. NF: 3.12 ± 0.54, *p =* 0.40) per 10 cm of total length of the main shoot compared to non-fertilised plants.

In *Juniperus communis*, in total, fertilised plants on nine analysed twigs had 6967 strobili in April 2017, 3137 of which were still present in July 2017 as developing cones, and resulted in a collection of 2374 matured cones in August 2018, whereas non-fertilised plants produced 747 strobili, 423 developing cones, and 397 matured cones. The mean number of strobili produced per 10 cm of twig (F: 13.27 ± 1.13 vs. NF: 2.25 ± 0.36, *p <* 0.0001), as well as the mean number of developing cones (F: 5.89 ± 0.36 vs. NF: 1.18 ± 0.31, *p <* 0.0001), and the mean number of matured cones produced per 10 cm of twig (F: 4.39 ± 0.29 vs. NF: 1.18 ± 0.31, *p <* 0.0001), were higher in fertilised when compared with non-fertilised plants.

**Figure 2:**
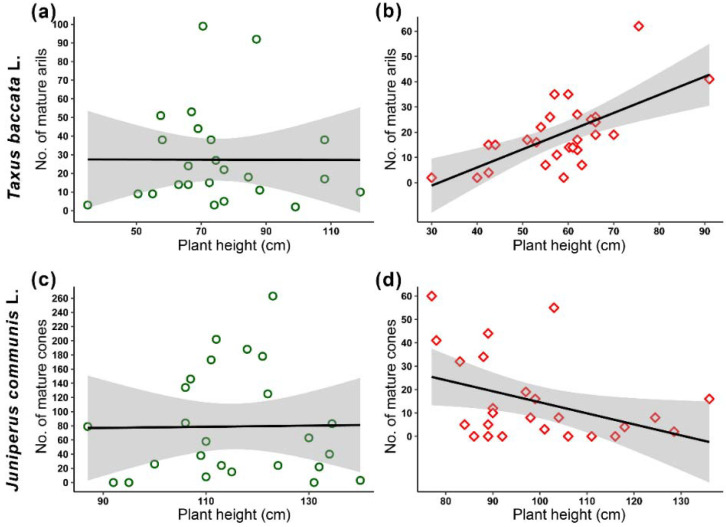
Number of *Taxus baccata* L. mature arils (**a**,**b**) and *Juniperus communis* L. mature cones (**c**,**d**) in relation to plant height (cm) in plants grown in fertilised (green, **a**,**c**) and non-fertilised (red, **b**,**d**) conditions.

There was no correlation between the number of mature arils or mature cones and plant height in fertilised plants of both species (Figure 2a,c); however, we observed that in non-fertilised *T. baccata*, the number of mature arils increased with plant height (y = −22.67 + 0.7229x, R^2^ = 0.424, *p =* 0.003, Figure 2a,b), whereas the opposite effect was observed in *J. communis* (y = −481 − 3.246x, R^2^ = 0.475, *p <* 0.001, Figure 2d).

#### 2.2.2. The Efficiency of Seed Production

In *T. baccata*, nutritional availability affected seed production efficiency; therefore, fertilised plants were characterised by a lower efficiency of female strobili developing into ovules (F: 48.07 ± 5.90% vs. NF: 62.39 ± 5.61%, *p =* 0.003) as well as lower efficiency of ovules developing into arils (F: 45.91 ± 5.84% vs. NF: 62.39 ± 5.51%, *p =* 0.046) and strobili into arils (F: 17.48 ± 3.80% vs. NF: 37.18 ± 3.58, *p =* 0.001) than non-fertilised plants.

In *J. communis*, nutritional availability affected the efficiency of seed production; thus, fertilised plants were characterised by a lower efficiency of female strobili development into developing cones (F: 60.24 ± 4.22% vs. NF: 40.69 ± 4.48%, *p =* 0.049) as well as into matured cones (F: 37.85 ± 2.51% vs. NF: 57.11 ± 2.97%, *p =* 0.001) than non-fertilised plants.

#### 2.2.3. Timing of Seed Maturation

**Figure 3:**
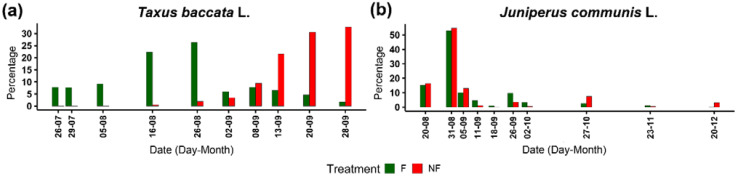
Percentage of mature (**a**) arils of *Taxus baccata* L. and (**b**) cones of *Juniperus communis* L. collected over time from female plants grown in different nutritional conditions (F—fertilised plants, green, NF—non-fertilised plants, red), concerning all collected generative structures.

In *T. baccata*, nutritional availability impacted the timing of seed maturation (Figure 3a). Almost 90% of seeds produced by fertilised plants started to produce mature arils around one month earlier compared to their non-fertilised counterparts in the analysed season.

In *J. communis*, too, nutritional availability impacted the timing of cone maturation (Figure 3b). In 2018, approximately 50% of cones were matured in both groups in August 2018; however, fertilised cones matured earlier; thus, in September 2018, 93.2% (2212/2374) of fertilised cones were already mature compared to 88.4% (351/397) for their non-fertilised counterparts.

### 2.3. Qualitative Features of Female Plant Reproductive Responses

#### 2.3.1. Seed Weight and Size

In *Taxus baccata*, nutrient availability impacted the weight of the seed, as fertilised plants produced heavier seeds (F: 55.55 ± 0.34 mg, range: 18.7–83.10 mg) compared to non-fertilised plants (NF: 51.73 ± 0.36 mg, range: 15.60–75.40 mg, *p <* 0.001). Additionally, morphological parameters such as curved seed length (F: 6.50 ± 0.014 mm, range: 5.32–7.66 mm vs. NF: 6.30 ± 0.022 mm, 4.53–7.86, *p <* 0.001) and curved seed width (F: 4.50 ± 0.010 mm vs. NF: 4.37 ± 0.009 mm, *p <* 0.001) and projected seed area (F: 22.44 ± 0.088 mm^2^ vs. NF: 20.85 ± 0.110 mm^2^, *p <* 0.001) were higher in seeds from fertilised plants.

In *Juniperus communis*, the weight of individual seeds ranged from 4 to 27.0 mg. Fertiliser availability impacted the weight of the seeds, as fertilised plants produced heavier seeds (F: 14.42 ± 0.18 mg, range: 6.1–26.90 mg) compared to non-fertilised plants (NF: 13.25 ± 0.16 mg, 4.0–21.10 mg, *p <* 0.001). Morphological parameters such as curved seed length (F: 4.92 ± 0.03 mm, range: 2.53–6.26 mm vs. NF: 4.51 ± 0.022 mm, 3.07–5.9, *p <* 0.001) and projected seed area (F: 9.62 ± 0.089 mm^2^ vs. NF: 8.57 ± 0.074 mm^2^, *p <* 0.001) were higher in seeds from fertilised plants; however, fertiliser availability did not impact curved seed width (F: 2.68 ± 0.019 mm vs. NF: 2.65 ± 0.018 mm, *p =* 0.25).

**Figure 4:**
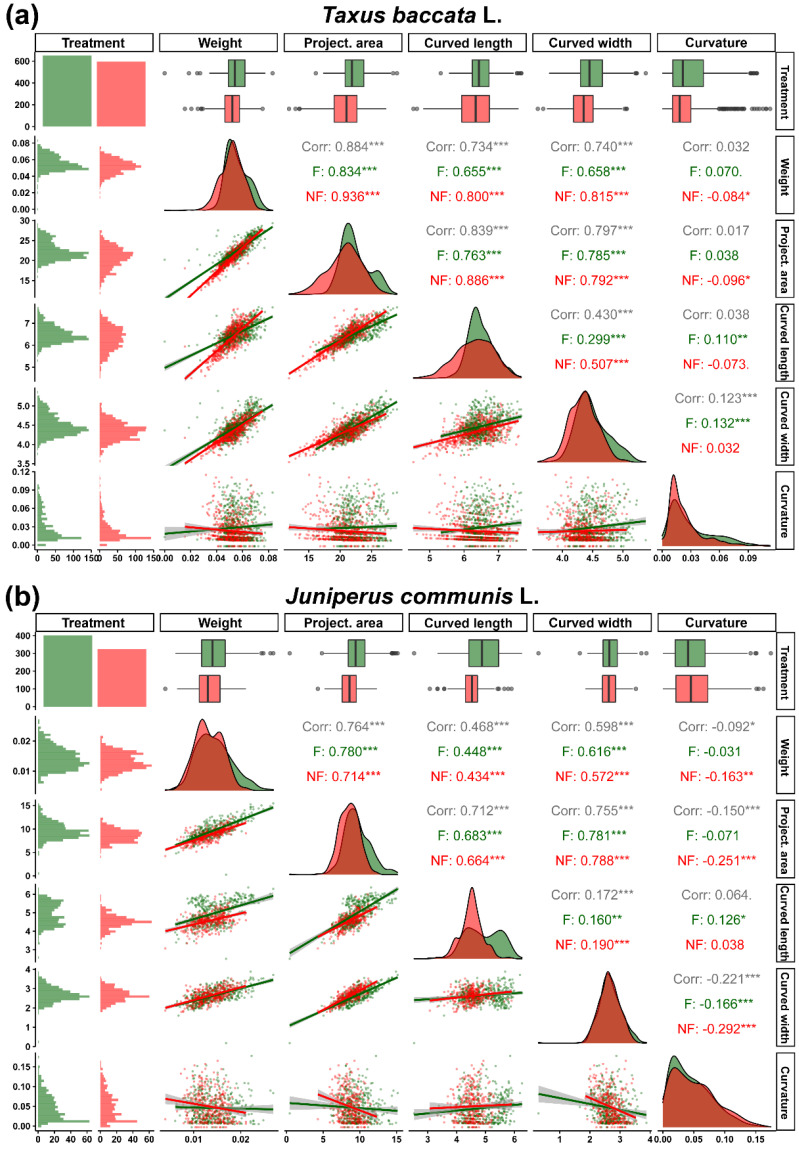
Correlation matrix and data distribution of seed weight (mg) and morphometric parameters (projected area, mm^2^; curved length and width, mm) of (**a**) *Taxus baccata* L. and (**b**) *Juniperus communis* L. seeds, collected from plants grown in fertilised (green) and non-fertilised (red) conditions. Stars indicate significant correlations (* *p* < 0.05, ** *p* < 0.01, *** *p* < 0.001).

We observed the impact of genotype (*p* < 0.001) and the interaction between genotype and fertilisation treatment (*p <* 0.001) on the weight and morphological parameters of seeds, and seeds from fertilised plants had higher mean values in each case. Correlations between the weight and morphological features of seeds were observed in both species, regardless of the treatment group (Figure 4a,b).

#### 2.3.2. Empty Seeds—Embryo Development

In 2017, in total 1903 mature cones were collected from *J. communis* plants. We observed up to five seeds produced per cone; however, only one cone produced five seeds in F plants, compared to none in NF plants. Furthermore, in F plants we observed 14 cones with four seeds, but only one cone in NF plants. Fertiliser did not affect the mean number of seeds per cone, thus the similar proportion of cones had three (F: 53.0% [901/1701] vs. NF: 54.10% [106/196]), two (F: 36.4% [619/1701] vs. NF: 38.3% [75/196]) or one (F: [166/1701] vs. NF: [14/196]) seed(s) in cones collected from plants from both treatment groups.

**Figure 5:**
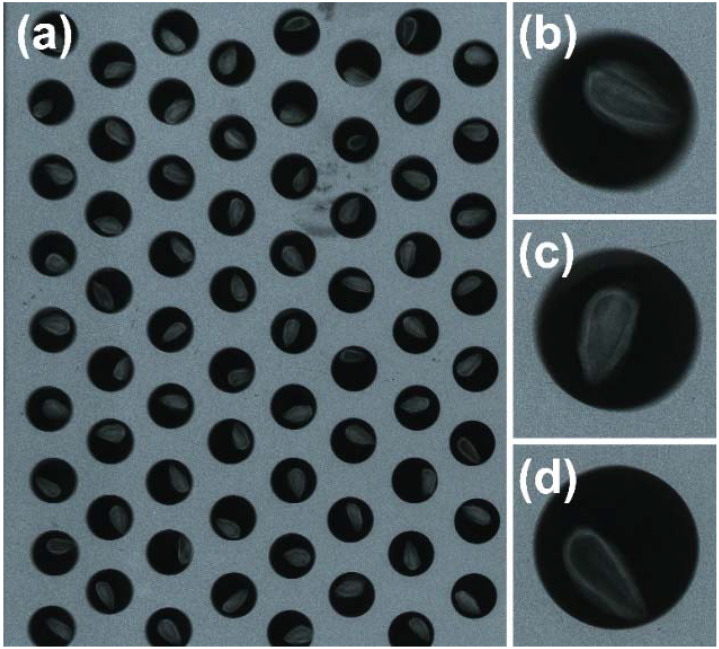
*Juniperus communis* L. dry seeds were analysed using the X-ray method for assessing viability. (**a**) X-ray film after analysis; (**b**) non-viable seed, with not properly developed embryo; (**c**) viable seed, a properly developed embryo with undamaged seed coat; (**d**) empty seed, coat without embryo.

Seed viability was described for 973 seeds collected in 2017 using the X-ray method (Figure 5a–d). Fertiliser availability did not affect the presence of embryo development thus a similar ratio of seeds with a properly developed embryo was observed in both treatment groups (F: 72.38% [401/554] vs. NF: 77.56% [325/419], *p =* 0.07). There was also a similar proportion of empty seeds (F: 25.63% [142/554], NF: 23.43% [86/419], and seeds with a non-properly developed embryo (F: 1.99% [11/554], NF: 1.91% [8/419]) in both treatment groups.

#### 2.3.3. Macronutrient Contents and Carbon-to-Nitrogen Ratio in Seeds

In *T. baccata*, application of fertiliser did not affect C content (F: 60.27 ± 0.32 vs. NF: 59.62 ± 0.32% dry mass, *p =* 0.18), N content (F: 1.38 ± 0.06 vs. NF: 1.30 ± 0.06% dry mass, *p =* 0.36) or C/N ratio (F: 44.28 ± 1.83 vs. NF: 46.07 ± 1.83, *p =* 0.50) nor N/P ratio (F: 5.33 ± 0.29 vs. 5.27 ± 0.29, *p >* 0.5). Fertiliser availability did not affect concentration (g/kg) of Mg (F: 1.596 ± 0.099 vs. NF: 1.419 ± 0.099, *p =* 0.24) and P (F: 2.501 ± 0.127 vs. NF: 2.495 ± 0.13, *p =* 0.97), but caused an increase of K (F: 2.750 ± 0.100 vs. NF: 2.374 ± 0.100, *p =* 0.028), and a decrease of Ca (F: 0.641 ± 0.069 vs. NF: 0.882 ± 0.689, *p* = 0.038) in fertilised *T*. *baccata* seeds.

In *J. communis*, application of fertiliser did not affect C content (F: 51.44 ± 0.25 vs. NF: 50.72 ± 0.23% dry mass, *p =* 0.055), but resulted in an increase in N content (F: 2.19 ± 0.06 vs. NF: 1.50 ± 0.05% dry mass, *p* < 0.001) and a decreased C/N ratio (F: 23.56 ± 0.93 vs. NF: 34.03 ± 0.85, *p* < 0.001) as well as an increase of N/P ratio (F: 7.03 ± 0.02 vs. 5.83 ± 0.16, *p <* 0.001) in *J. communis* seeds. Fertiliser availability did not affect concentration (g/kg) of Mg (F: 1.596 ± 0.099 vs. NF: 1.419 ± 0.099, *p = 0.24*), but caused an increase in P (F: 3.222 ± 0.110 vs. NF: 2.548 ± 0.110, *p =* 0.003) and K (F: 7.391 ± 0.379 vs. NF: 5.517 ± 0.379, *p =* 0.008), and a decrease in Ca (F: 2.452 ± 0.583 vs. NF: 4.561 ± 0.583, *p =* 0.033) in fertilised *J. communis* seeds.

#### 2.3.4. Metabolite Profile in Seeds

**Figure 6:**
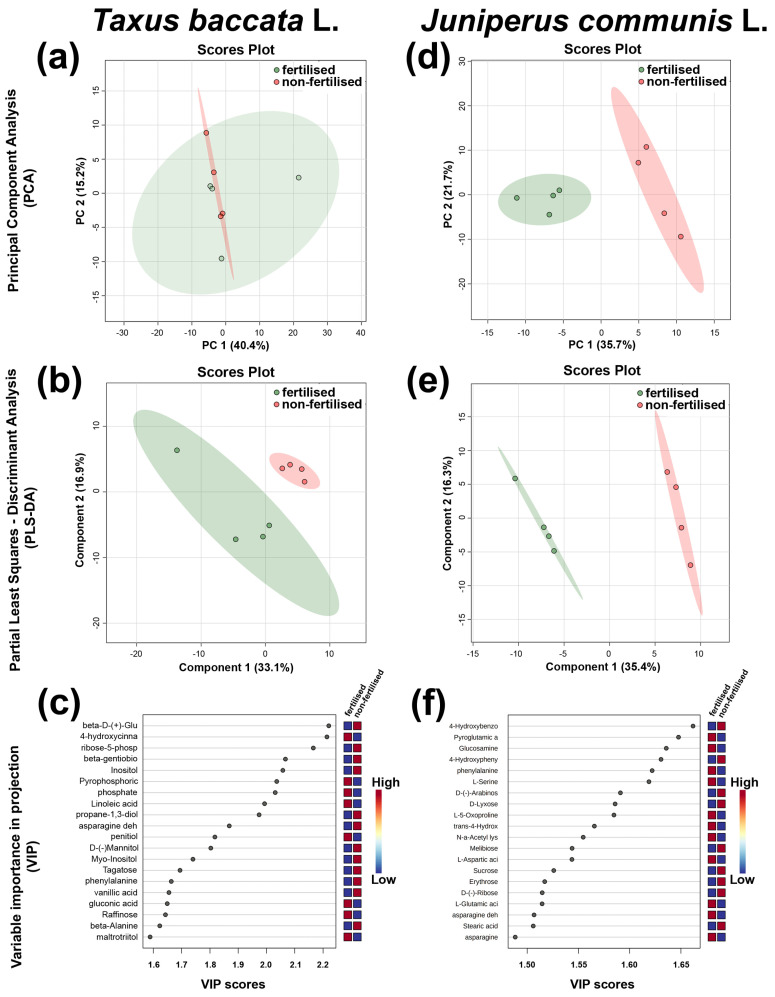
Results of the metabolome data analysis. (**a**,**d**) Principal Component Analysis scores—plot between the selected Principal Components (PC), and (**b**,**e**) Partial Least Squares–Discriminant Analysis (PLS-DA)—plot between the selected Principal Components (PC) showing separation between fertilised and non-fertilised dry seeds collected from *Taxus baccata* L. and *Juniperus communis* L. plants, grown in fertilised (green) and non-fertilised (red) conditions. The explained variances are shown in brackets; (**c**,**f**) VIP scores showing the top 20 important metabolites that contribute the most to the PLS-DA plots of seeds. Colours in the variable importance in projection plot represent relative intensities, where red and blue symbolise higher and lower values, respectively.

Seeds of fertilised and non-fertilised plants were randomly selected to investigate the impact of the environment on metabolite profile in relation to seed quality. A nontargeted metabolomics strategy GC/MC was used to detect primary metabolites. Stable intensities were detected for the internal standards in each sample. Quality control (QC) samples clustered together in the centre of the PCA scores. All of this suggests that the data acquisition was reproducible and robust. Overall, we extracted 194 metabolites from the matrix via GC-MS (Supplementary Table S1). We performed unsupervised PCA and Partial Least Squares-Discriminant Analysis (PLS-DA) to determine the variations in metabolites between different treatments individually for each species. PC1 and PC2 explained 55.6% and in *J. communis* 57.4% of the total variation in *T. baccata* and *J. communis*, respectively, and a clear separation between the treatment groups was observed only for *J. communis* (Figure 6a,d). Analysis by PLS-DA explained approximately 50% of the total variation; however, the treatment groups were also separate (Figure 6b,e). To identify potential variables, we screened the differential metabolites by comparing the fold change (−1.1 < FC > 1.1), *p*-value (<0.05), and variable importance in projection (VIP) score (>1) of the metabolites in each pair of comparisons (Table 1, Figure 6c,f).

In *T. baccata*, we did not identify differential metabolites among the 194 present within the sample. Only one metabolite, ribose-5-phosphate (*p* = 0.04), could be considered significantly different (based on *p*-value), but it could not be classified as a differential metabolite according to other values.

**Figure 7:**
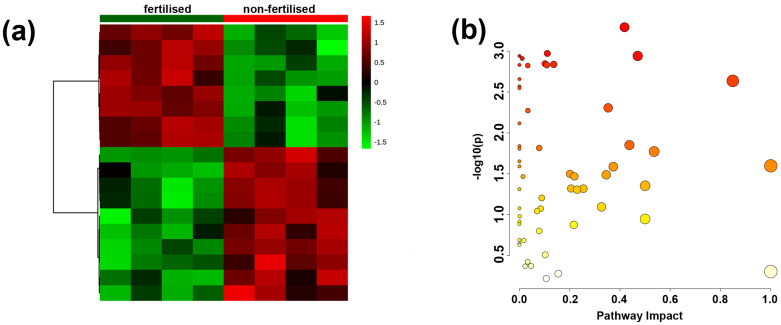
Results of the metabolome data analysis of dry seeds collected from *Juniperus communis* L. plants grown in fertilised (green) and non-fertilised (red) conditions. (**a**) The clustering result is shown as a heatmap (distance measure using correlation, and clustering algorithm using ward.D); colours represent metabolite relative intensities with red and green symbolising the highest and lowest values, respectively. (**b**) Metabolic pathway analysis plot created using MetaboAnalyst. Plot depicts several metabolic pathway alternations induced by long-term fertiliser availability. The *x*-axis represents the pathway impact value computed from pathway topological analysis, and the *y*-axis is the –log of the *p*-value obtained from pathway enrichment analysis. The pathways that were most significantly changed are characterised by both a high –log(*p*) value and a high impact value (top right region).

In *J. communis*, we identified 18 differential metabolites among the 194 present within the sample (Table 1, Figure 6 and Figure 7). Fertilised plants had a higher accumulation of some metabolites (e.g., asparagine (dehydrated), pyroglutamic acid, L-aspartic acid, glucosamine, L-serine, L-5-oxoproline) as well as a lower amount of others (e.g., erythrose, stearic acid) (Supplementary Figures S1 and S2). Moreover, several pathways were impacted in fertilised plant seeds (Figure 7b, Supplementary Table S2).

## 3. Discussion

Five interesting insights can be derived from our study. First, the response of reproductive output to the changed environment (higher nutritional availability) did not differ between species according to their life history, resulted in more numerous production of female reproductive structures, and was accompanied by higher vegetative growth. Second, the higher productivity of female flowers (strobili) was accompanied by a higher total number of seeds, but also by a lower efficiency of seed production. Third, the environment impacted both production (seed-set and fruit-set) and quality of seeds; thus, fewer seeds produced by plants did not mean a higher seed mass. Fourth, species life history is related to the reproductive response to fertilisation of dioecious woody plants. Fifth, environment rather than genotype impacted the timing of plant and seed maturation, and plants can enhance seed quality by increasing the time needed for seeds to mature.

**Figure 8:**
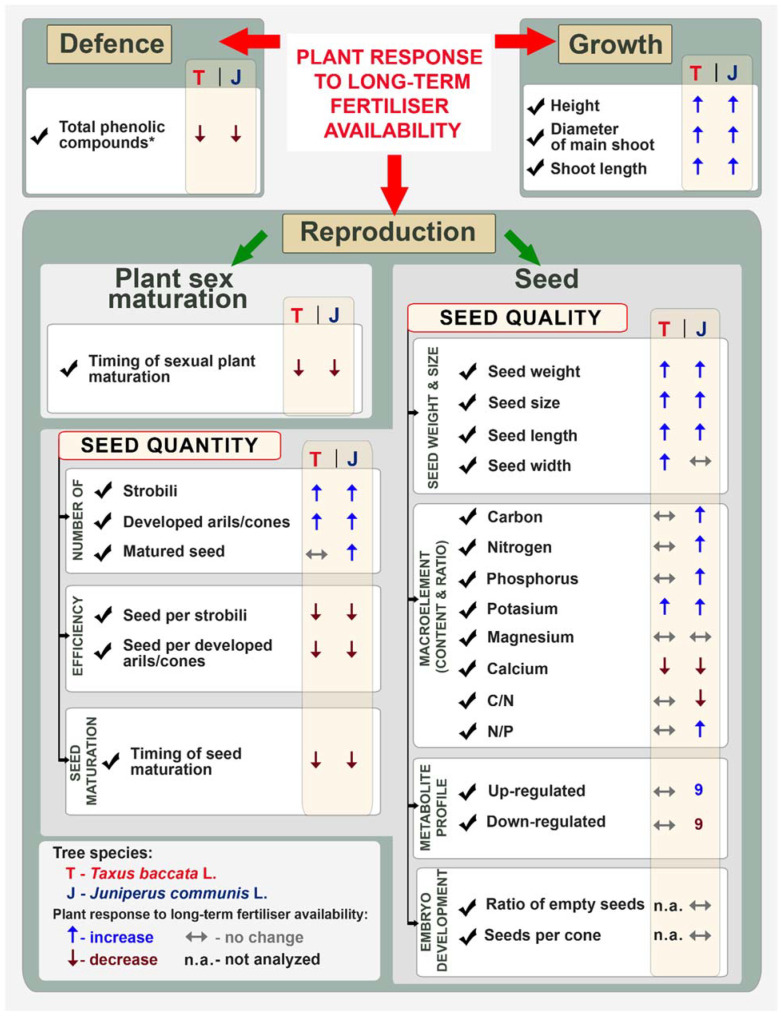
Plant response to long-term fertiliser availability. * Data summarised after [31,41].

The life cycle of flowering plants can be considered as a succession of distinct growth phases (e.g., vegetative and reproductive phases) that are triggered and modulated by both environmental and endogenous stimuli, and are under the control of a complex genetic network [45]. Juvenile-to-adult phase transition indicates that the plant has acquired reproductive competence. Moreover, the principle of allocations suggests that increased nutrient allocation to reproduction will result in costs such as reduced growth and survival [2]. We observed that the fertilised plants had allocated available resources for vegetative growth; thus, fertilised plants of both species were taller, had a wider diameter of the main shoot, and had longer branches than non-fertilised ones. Resource availability impacted the time of plant maturity; thus, a higher proportion of fertilised plants reached sexual maturity earlier and produced strobili in both species. However, this phenomenon was more noticeable in the case of the pioneer species *J. communis*. The proportion of mature plants was approximately twice that of non-matured ones. This is true especially for pioneer species, because a plant that reproduces early would succeed in the colonisation of new habitats if individuals produce offspring before their accidental death [46]. Fertilised plants of *T. baccata* produced a higher mean number of strobili (4× higher) and ovules (2×), but a similar number of arils per plant (F: 27 vs. NF: 19); however, fertilised *J. communis* plants were characterised by a higher number of strobili (6×), developing cones (5×), and matured cones produced per 10 cm of branches (3.7×) compared to non-fertilised ones. Our results are framed within the theory of costs of reproduction, thus there is a trade-off at the physiological level of resource allocation to vegetative growth, reproduction, or defence [2] (Figure 8). These point to the presence of different strategies for resource allocation in *T. baccata* and *J. communis*. Non-fertilised individuals of a *T. baccata* allocated more to reproduction, such that only two times fewer mature arils were accompanied by a ~40% decrease of vegetative growth, whereas pioneer *J. communis* produced five times fewer cones, accompanied by only a 30% decrease in vegetative growth. These differences can also result from the different times needed for seed development in the two species [26,35].

Seeds are “genetic time capsules” provided by the mother plant and assigned to ensure plant fitness. Upon fertilisation, seed development and maturation are fundamental processes controlled by the genetic make-up of the mother plant, but also influenced by the environment it experiences. Therefore, the main female plant function is to produce seeds that will be able to germinate and produce viable seedlings, so that both the quantity and the quality of seeds should be examined simultaneously. In our study, fertilisation increased the total number of seeds, and thus more cones were produced by fertilised plants of both species. Moreover, fertilisation did not affect the mean number of seeds produced per cone for *J. communis* as well as embryo development; thus, a similar proportion of properly developed embryos was noted during X-ray scanning in both treatment groups (~70%). Our results are in agreement with the results of a long-term N fertilisation study related to the reproductive ecology of red oaks, in which N addition also increased acorn production (up to nine-fold) [43]. However, when success in seed production was taken into account in our study, fertilised plants of both species lost the competition with non-fertilised plants. Non-fertilised plants were characterised by a higher ratio of strobili that developed into seeds. Plants commonly produce more ovules than seeds, and environmental conditions as well as pollen availability can alter the reproductive success of plants by ovule attrition [44]. Taking this into consideration, we kept plants in the same environmental conditions and kept female plants close to male plants (in a 1:1 ratio) to ensure a high probability of pollination and fertilisation. This suggests that, although in a rich environment, female plants invest more into production of numerous flowers, while the efficiency of seed development and seed production is related to factors other than just nutritional availability and pollen availability [47]. Moreover, our earlier studies showed that fertilised female plants of both species are characterised by higher photosynthetic activity [38,48] and higher biomass [39] than their non-fertilised counterparts. These observations indicate that the allocation of plant resources to seed production is a reasonable/controlled investment of resource allocation; thus, in a non-fertilised environment, when the plant has already invested part of the available resource for female reproductive structure development, the necessary resources were provided by the plant (at the cost of vegetative growth or resource allocation) to ensure development of seeds [47]. All of the above suggests that plants have evolved mechanisms of compensation of cost of reproduction, and this mechanism can be switched on after flower production but before embryo/seed development, and results in abortion of female reproductive structures just shortly before or after ovule fertilisation.

Seed size is described as a potential quality marker of seedling vigour; thus, heavier seeds are often used for seedling production [49], although this is not always the case [50]. Moreover, it is assumed that when resources are scarce, plants produce heavier seeds [51]; however, both increases and decreases in seed mass have been observed under stressful conditions [5]. In our study, female fertilised plants produced more seeds that were heavier and bigger than non-fertilised counterparts. However, inconsistency within the data distribution of seeds’ morphometric features and a shift between fertilised and non-fertilised plants was observed; thus, longer seeds were frequently observed in fertilised *T. baccata* (3.1%), whereas in *J. communis*, longer seeds were produced by non-fertilised plants (8.6%). We observed an increase in seed weight in fertilised conditions (*T. baccata*—by 7.38%, *J. communis*—by 8.8%) although numerous seeds were produced. However, it is hard to compare the obtained results with other studies because similar previous research was done mostly on annual and perennial plants, not woody plants [21]. N addition in the maternal environment increased seed production of *Potentilla tanacetifolia* by 148%; however, it significantly decreased seed weight at the same time. Our studies are in agreement with those performed on *Pinus pinaster* seeds [5], where seed weight similarly decreased as the environmental conditions became more stressful. Although heavier seeds potentially equate with more resources available to seedlings, heavier seeds of English yew do not always germinate better, and produce seedlings with better vigour than lighter ones. Additionally, as previously suggested, heavier seeds may contain an inhibitor of seed germination [52]. Recent studies also show that there is a problem of germinability of *J. communis* in which efficiency of seed germination is very low, but there is lack of information regarding seed weight or size [53,54].

Fertilisation did not impact C, N, Mg, and P contents or C/N ratio in *T. baccata* plants; however, in the pioneer species *J. communis*, an increase in N (by 60%) and P (by 30%) content as well as a decrease in C/N ratio (by 44%) was observed, but C and Mg contents did not change. Moreover, higher nutritional availability, in the same manner, shifts the content of K (increase) and Ca (decrease) in *T. baccata* and *J. communis* seeds. Higher doses of fertiliser could lead to an increase in soil acidification and, thus, a lower uptake of Ca from the soil was observed. However, soil acidification should also be related to a decrease in K, P and Mg [12], which was not true in our studies.

Recent studies have shown a negative effect of climate warming and atmospheric deposition on the viability of *J. communis*, which is also related to impacts on the nutrient status of the plant [26,55]. In these studies, the C/N ratio ranged between 23.88 and 61.38 in needles. Additionally, our previously published data showed that fertilisation affected the C/N ratio in needles, with fertilised females having a C/N ratio that was half that of non-fertilised plants [41]. *J. communis* are characterised by different C/N ratios between needles and seeds; thus, fertilised plants produced seeds with a higher C/N ratio compared to needles, whereas non-fertilised had a lower C/N ratio. Moreover, differences in resource availability did not result into changes of the metabolite profile of *T. baccata* seeds; however, some of the metabolites were down-regulated and some were up-regulated in seeds of *J. communis*. Fertilised *J. communis* plants allocate N resources mainly to amino acids; thus, an at least two-fold increase in asparagine, aspartate, lysine, phenylalanine, serine and proline was observed. Moreover, we also observed decreases in many monosaccharides in seeds from fertilised plants. Both an increase of amino acids and the decrease of carbohydrates are related to N metabolism; thus, carbohydrates provide a carbon skeleton and energy for N assimilation [56,57]. Moreover, a decrease of erythrose can point to an increase in aromatic amino acids synthesised via the shikimate pathway [58]. High contents of N and other macronutrients as well as high levels of N compounds, together with the high level of proline in seeds, point to the mechanism of plant detoxification and/or N storage in seeds. N allocated in seeds can be further used during seed germination and seedling growth [59], which could enhance seedling growth of a pioneer species in poor environments and soil conditions.

Growth, defence, and reproduction are linked together by a constraining relationship, and plants need to allocate available resources by compromising between those three main life history traits [60]. In dioecious plants, many previous reports point to sexual spatial segregation of both sexes [25,61], which is related to a higher cost of reproduction of female compared to male plants [2]. Thus, males produce generative structures and pollen grains, whereas females not only produce flowers, but also invest in the developing embryos, providing them with reserves and producing features needed for seed dispersal [2]. Therefore, female plants allocate their resources into vegetative growth, defence, and reproduction; however, female individuals of long-lived plant species have to compensate for all those costs not only once per year, but also between years [62,63,64]. Different reproductive costs are the main cause of sexual secondary dimorphism, which was previously observed in dioecious species [65,66]. This suggests that females of woody plants evolved mechanisms of efficient allocation of available resources for reproduction, that allows them in (or restricts them to) habitats where they can best meet the higher cost of reproduction [2]. Therefore, both seed quantity and quality, related to the fitness of each offspring, are important traits of the reproductive response of female plants. One of the most fundamental life history trade-offs is between the size and the number of offspring produced by individuals. According to the first model of resource allocation, the optimal investment per offspring depends on the relationship between the amount of investment per offspring and offspring fitness. The authors have concluded that the optimal investment per offspring was independent of the size of the resource pool available for investment in the offspring [67]. However, plants invest in not only one but many different resources in seeds, and therefore, seed quantity and quality may be influenced by more than one resource. This was inserted into the multiple resources model, where the optimal balance between size and number of offspring, which predicts that the optimal allocation will depend on the pool of available resources [68] and growth conditions [69]. According to the resource allocation theory, resources directed toward reproduction should balance the cost related to the number of seeds produced as well as their quality. The optimal size of seeds is related to the optimal allocation of C and N, which translates into an appropriate C/N ratio in the seeds produced and appropriate seedling fitness. According to the model, the optimal seed mass C allocation is positively related to the C/N ratio, because the optimal carbon-based compounds increase as C/N ratio increases. The optimal investment of N-based compounds, absolute seed N content, is negatively correlated with the C/N ratio [68]. Therefore, with the optimal C/N ratio, the C and N contents in seeds should be stable and ensure optimal carbon-based energy storage compounds, carbohydrates and lipids, as well as optimal levels of N-based compounds, proteins and amino acids [68]. All changes in C and N content will be related to changes in seed weight. The life history of the analysed species also points to different responses to environmental resources; juniper as a pioneer of N-poor habitats had a strategy for long-term N-storage by females, but *T. baccata,* preferring richer, late successional habitats, did not show such an adaptation [7]. These differences between rich- and poor-habitat species confirmed that *J. communis* has different strategies of resource management from those of *T. baccata*. Differentiated N, P and K contents between treatment groups of *J. communis*, but not *T. baccata*, confirm different strategies of resource storage and use by female plants, which is in line with other studies [41,70]. Further studies are needed to describe the impacts of the nutritional status of maternal plants on seed germination and seedling vigour in relation to different markers of seed quality.

## 4. Materials and Methods

### 4.1. Species Studied

We focused our study on female plants of two dioecious species: *Taxus baccata* L. and *Juniperus communis* L. var. *communis*. These species are dioecious, wind-pollinated coniferous shrubs or trees [35,36]. They are characterised by different habitats: *T. baccata* can grow typically on humus and base-rich soils [71], whereas *J. communis* is a typical shrub species of poor soil and harsh environments [72].

Macrostrobili (female reproductive organs, female flowers) of *T. baccata* are 1.5–2.0 mm long, located singly or in pairs in leaf axils on the underside of shoots, not forming cones, with a single ovule. From our observation in Poland female strobili develop from late summer/early autumn of the year before fertilisation, then pollination occurs in March-April the next year, followed by fertilisation approximately in June/July, and mature seeds can be collected during late spring–autumn. Seeds are ovoid, smooth and shiny, brown-yellow, 6–7 × 5 mm at maturity, with a tough seed coat, partly surrounded by a fleshy red aril, typically 9 × 7 mm which falls with the seed at maturity, with ‘fruit’ ripening in the first year (after [35]).

Macrostrobili of *J. communis* are approximately 2 mm and green when young, 5–10 mm and globose or rather longer than broad, surrounded at the base by minute scale-like bracts, persisting when mature. A true cone, although the 3–16 scales do not become hard and woody but remain fleshy, is fused and berry-like, and black with a blue bloom when ripe. Seeds are elongated, not winged, ovoid, three cornered, and with grooves and resin pockets over the whole seed, one seed per cone-scale 1–3 (max. 6) seeds per cone, each 4–5 mm long, embedded in the resinous, mealy pulp and retained within the cone for dispersal. Female cones ripen during the second or third year [36].

### 4.2. Experimental Design

The experiment was conducted at the Institute of Dendrology of the Polish Academy of Sciences in Kórnik, Poland. Rooted shoots (50) of 10 female trees of both species were used in the experiment. Plants were produced as described in previous publications [24,25,40]. The experiment was conducted on plants grown under two levels of nutrient conditions since half of the plants received an application of fertiliser each year from March 2013 to 2017. Osmocote Exact 5–6 M (ICL, Tel Aviv, Israel) fertiliser was applied at the recommended dose of 6 g/L and the fertilised plants received 0.75 g N (N-NO_3_—0.33 g, N-NH_4_—0.42 g), 0.45 g P_2_O_5_, 0.6 g K_2_O, 0.125 g MgO, and microelements (22.5 mg Fe, 3.0 mg Mn, 1.0 mg B, 2.5 mg Cu, 1.0 mg Mo, 0.75 mg Zn) per litre of soil. The control group of non-fertilised plants was grown without the use of any fertiliser or other supplements.

Each pot was irrigated separately with an automatic irrigation system. As fertilisation increases the growth of the plant and is related to water availability, plants from both groups were irrigated with different amounts of water; thus, fertilised plants got 1.25× the volume of water as non-fertilised plants during irrigation, and plants were watered to keep a medium level of soil moisture during the whole vegetation season. In March 2013, rooted cuttings of both species were placed in five-litre pots and then transferred to ten-litre pots in May 2016. This transfer was done to minimise the negative effect of pot size on plant growth [46].

Female plants from both species (*T. baccata* L. [*n* = 50] and *J. communis* [*n* = 52]) without any visible signs of flowering were randomly selected and tagged from each of the two groups in March 2013, and subsequently monitored for the presence of female flowers and seed development. An equal number of plants from the fertilised and non-fertilised groups and derived from the same parental material were used in the analysis. In 2015, six individuals of *T. baccata* were removed from the fertilised group as they were injured by an early frost. Plants were grown in the presence of male plants of both species to increase the probability of pollination.

**Figure 9:**
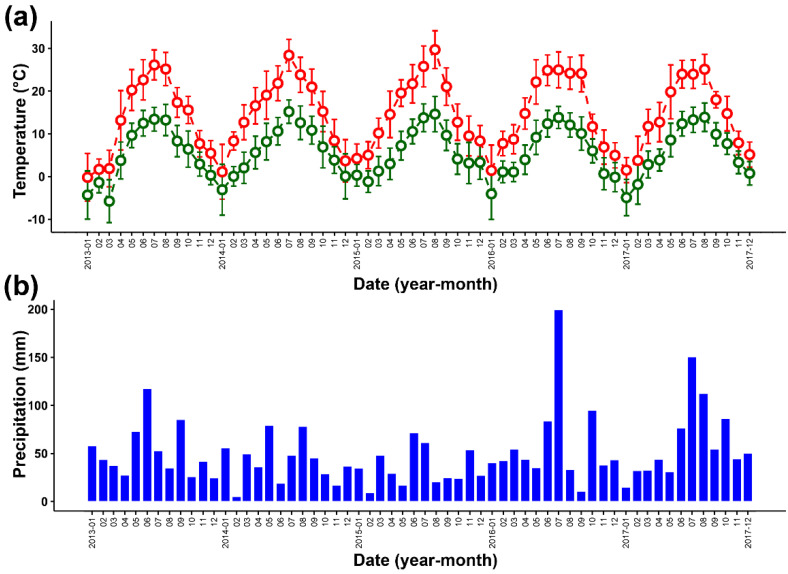
Meteorological conditions during the period of seed development. (**a**) Monthly maximum (red) and minimum (green) mean values of air temperature (°C). (**b**) Monthly mean of the total precipitation (mm).

Weather and soil conditions: During the study, measurements of air temperature were recorded each hour using four EL-USB-2+ data loggers (EasyLog, Inc., Salisbury, UK) and the amount of precipitation was recorded three times per day (at 6 a.m., 12 a.m., and 6 p.m.). Monthly maximum and minimum values of air temperature and the sum of precipitation during the period of seed development were similar to long-term averages in this region of Poland (Figure 9).

The main soil in pots used at the beginning of the experiments contained following concentrations (in g/kg) of elements for *T. baccata* and *J. communis*: 36.0 ± 0.9 total C, 2.0 ± 0.4 total N, 0.3 ± 0.06 total P, 0.46 ± 0.05 total Ca, 0.84 ± 0.13 Mg, and 250.0 ± 25.0 mg/kg Na. Soil samples were analysed by JARS S.A. (Mysłowice, Poland) according to the Polish Committee for Standardization (analysed PKN, 2012, 2009, 2002).

In September 2016, *T. baccata* soil samples were collected from randomly chosen pots of non-fertilised and fertilised plants and their chemical composition was analysed. Soil of fertilised (F) and non-fertilised (NF) plants contained the following concentration (in g/kg) of the elements, respectively: total C (F: 35.7 ± 2.0 and NF: 34.7 ± 2.0), total N (F: 1.8 ± 0.15 and NF: 1.4 ± 0.07), C:N ratio (F: 19.4 and NF: 24.2), total P (F: 0.30 ± 0.01 and NF: 0.11 ± 0.01), total Ca (F: 0.51 ± 0.02 and NF: 0.55 ± 0.01), Mg (F: 0.33 ± 0.04 and NF: 0.26 ± 0.01), and Na (mg/kg, F: 261.00 ± 18.4 and NF: 286.3 ± 8.7). Whereas, *J. communis* soil samples from randomly chosen pots of non-fertilised and fertilised plants were collected in September 2017. Soil of fertilised (F) and non-fertilised (NF) plants contained the following concentration (in g/kg) of the elements, respectively: total C (F: 40.4 ± 1.2 and NF: 42.3 ± 0.7), total N (F: 1.8 ± 0.12 and NF: 1.7 ± 0.06), C:N ratio (F: 22.4 and NF: 25.1), total P (F: 0.39 ± 0.05 and NF: 0.15 ± 0.02), total Ca (F: 0.69 ± 0.02 and NF: 0.53 ± 0.06), Mg (F: 0.38 ± 0.04 and NF: 0.33 ± 0.02), and Na (mg/kg, F: 303.67 ± 3.84 and NF: 257.7 ± 10.6).

### 4.3. Plant Morphology and Sexual Maturity

Each year, the growth of female plants was monitored. Plant growth was measured in December, after annual growth had stopped. Measurements were taken from the top of the soil to the top of the growth meristem. Moreover, each plant was characterised by the diameter of the main shoot measured with an electronic vernier caliper (Aimometer) at the end of the experiment (*T. baccata* in 2016 and *J. communis* in 2018).

Plants were observed from 2013 through 2016 (*T. baccata*) or 2017 (*J. communis*). Flowering of *T. baccata* plants occurs in Poland in March each year, and at this time plants were described by the presence or absence of female flowers, and all branches were checked. Similarly, we observed the presence or absence of female flowers in *J. communis*; however, these plants were monitored in late spring, as flowering of *J. communis* female plants in Poland occurs at the end of April though into the beginning of May. This was repeated in summer, due to the high probability of miscounting lateral growth meristem instead of female flowers during flowering time when counting directly on the shoots without a microscope. It should be emphasised that in *J. communis* the mature seed develops mostly within a three-year cycle [37]; initiation of reproductive buds starts in autumn, pollination and pollen germination take place the next spring, and subsequently the female gametophyte starts to develop, followed by fertilisation and ripening of the embryo and megagametophyte; then, finally, the embryo is mature and ready for dispersal mostly in the third year. Because of this, plants that in May/June 2016 were characterised by the presence of at least one developing female gametophyte were classified as being already sexually mature in 2015. Each year, the ratio of sexually mature individuals was calculated as the number of individuals with the presence of cones as a proportion of the number of all individuals.

### 4.4. Quantitative and Qualitative Features of Female Plant Reproduction

#### 4.4.1. Number of Female Flowers and Matured Arils/Cones

The efficiency of reproductive efforts of *T. baccata* female plants was expressed by the number of female strobili produced and noted in 2016; thus, we observed some losses of the mature arils caused by animal activity in 2015. For this purpose, we counted the number of ovules in March 2016, the number of developing arils in June 2016, and the number of collected mature arils in 2016. Moreover, the number and length of all main branches as well as the total height of the plants from ground level to the apical meristem were measured in September 2016.

In *J. communis*, the observation was made within two seed development cycles: the first between 2015 and 2017, and the second between 2016 and 2018. Within the first cycle, we counted all female strobili/initiated cones present on the branches in June 2016 and all matured cones in September 2017; however, within the second cycle, due to the tall crowns of fertilised plants, we selected and marked three branches at three levels of the plant’s main shoot (⅓, ½ and ¾ of total plant height). At the end of April 2017, we counted all flowers present on the branches and divided them into two groups according to size and colour, as follows: improperly developed (small and yellowish with brown sac-like structure) and properly developed (bigger green cones with signs of female gametophyte development). We counted the number of developing cones monthly from April 2017 to September 2017 and from March 2018 to December 2018. The quality of collected cones was described by the presence or absence of damage done by predators or fungi, in accordance with McCartan and Gosling [73]. Moreover, we measured the total height of the plants from ground level to the apical meristem in September 2016 and September 2017. In 2018, each plant was characterised by the diameter of the main shoot measured with an electronic Vernier calliper (Aimometer), the length of selected branches, and the total height of the plants from the level of the ground to the apical meristem.

#### 4.4.2. Seed Production and Timing of Arils/Cones Maturation

*T. baccata* seeds were collected in 2016. The mature aril of *T. baccata* produces one seed; therefore, the number of mature arils equals the number of collected seeds. In 2016, all mature arils present on the *T. baccata* plants were separately collected in approximately one-week intervals from July to November 2016. Collected arils were stored at 4 °C and seeds were collected from them within two weeks. The timing of aril maturation was described as a percentage of the number of mature arils collected at a given sampling time in relation to the number of all collected arils.

*J. communis* seed production was described for 2017 and 2018. Mature cones of *J. communis* can have between one and six seeds, and the number of cones does not equal the number of seeds; therefore, we counted the number of seeds per individual cone from all cones collected in 2017 and from 30 cones per plant in 2018, and the mean number of seeds per cone was used to estimate the total number of seeds collected from plants in 2018. In *J. communis*, not all non-fertilised plants were sexually mature in 2015; thus, the time of maturation of *J. communis* cones was described on the basis of observation of female flowers produced in 2016, pollinated in 2017 and matured cones in 2018. Cones were classified as mature in accordance with McCartan and Gosling [73] and collected in approximately one-week intervals from July to December 2018. Collected cones were stored in room temperature and seeds were collected from them within two weeks. Similarly, timing of cone maturation was described as a percentage of number of mature cones collected within the sampling point in relation to number of all collected cones.

#### 4.4.3. Seed Weight and Morphometrics

To characterise the seed weight of *T. baccata* and *J. communis*, all seeds collected from the same maternal line were pooled together within the treatment group. Seeds were air dried, and when they contained approximately 10% water content, they were individually weighed. Each seed extracted from *T. baccata* matured arils (in 2016) and from *J. communis* matured cones (in 2017) was individually weighted. After weighing, they were individually analysed using WinSEEDLE™ Analysis system for seeds (Regent Instrument, Québec, QC, Canada). Therefore, each seed was characterised by the following features: seed mass, projected area, curved length, curved width, and curvature.

#### 4.4.4. Embryo Development

*J.communis* L., but not *T. baccata* L., is characterised by production of numerous empty seeds; therefore, we analysed the viability of seeds described by the presence of an embryo only within the *J. communis* seeds and assumed that all *T. baccata* seeds contained an embryo. We selected 977 seeds (557 fertilised and 420 non-fertilised) from all seeds of *J. communis* collected in 2017 and analysed them with X-ray. X-ray scanning was performed in Leśny Bank Genów Kostrzyca, Miłków, Poland, in accordance with Załęski et al. [74]. Seeds were proceessed individually and information regarding their treatment group and maternal tree were recorded. The viability of seeds was determined via the X-ray method without contrast using the FAXITRON-RAY Systems 43588A X-ray machine. Seeds were classified according to embryo development into three classes: (1) not viable (improperly developed embryo, Figure 5b), (2) viable (properly developed embryos with undamaged seed coat, Figure 5c), and (3) empty (lack of embryo, Figure 5d) seeds.

#### 4.4.5. Macronutrients Analysis and Carbon-to-Nitrogen Ratio

Dry seeds collected from 10 plants of both species grown in fertilised and non-fertilised conditions were used to determine the C/N ratio (*n* = 40). Dry ground seeds (4 mg) were analysed separately from individual samples. C and N content (% of dry mass) was determined using an elementary analyser (CHNS-/2400 Series II (PerkinElmer, Waltham, MA, USA). The percentage of P, K, Ca, and Mg in the samples was determined using an inductively coupled plasma time-of-flight mass spectroscope (ICP–TOF-MS; GBC Scientific Equipment; Braeside, Australia). Prior to analysis, samples were mineralised in nitric acid. Calibration was conducted by the external standard method according to the standard PN-EN ISO 11885:2009 [75].

#### 4.4.6. Metabolites

Recently published extraction protocols [76,77] were followed for preparing samples for GC-MS analysis. Briefly, the seed sample (200 mg) was weighed and ground into fine powders in liquid nitrogen. Next, 1 mL of ice-cold methanol: chloroform solution (3:1, *v*:*v*) was added. Samples were then vortexed at 6000 rpm for 15 s (repeated three times), after that, samples were centrifuged at 15,000× *g* for 10 min, and 300 µL of the supernatant from each sample was transferred to a new vial and vacuum-dried at room temperature.

For derivatisation, 80 µL of 20 mg/mL methoxyamine hydrochloride (dissolved in pyridine) was added to the vial, and the samples were then transferred into an oven and incubated at 37 °C for 1.5 h. Then, 80 µL of MSTFA (N-methyl-N-(trimethylsilyl)trifluoroacetamide) was added, followed by incubation at 37 °C for 1 h. Supernatants of all samples were mixed, vacuum-dried, and derivatised in the same manner to prepare QC samples. 1 µL of the derived extract from each sample was analysed using a TRACE 1310 GC oven with TSQ8000 triplequad MS from Thermo Scientific (Waltham, MA, USA) coupled with a DB-5MS capillary column (30 m × 0.25 mm × 0.25 µm, J&W Scientific, Fossen, CA, USA). Chromatographic separation conditions in gradient mode were kept as follows: 70 °C for 2 min, followed by 10 °C/min up to 300 °C, then 300 °C for 10 min. A PTV injector was used for sample injection with a temperature gradient from 40 to 250 °C, the column interface was maintained at 250 °C and the source temperature at 250 °C. The ion source was operated in the m/z range of 50–850 in EI positive mode, the electron energy was set to 70 eV and the carrier gas (He) flow rate was 1.2 mL/min. The mixture of Supelco C7–C40 saturated alkanes standard was run prior to the samples to calculate the retention index of each feature. The QC samples were run at the start, middle, and end of the analysis (every 5 samples).

Raw MS-data were converted to abf format and analysed using MSDial software package v. 3.96. To eliminate the retention time (Rt) shift and to determine the retention indices (RI) for each compound, the alkane series mixture (C-10 to C-36) was injected into the GC/MS system. Identified artifacts (alkanes, column bleed, plasticisers, MSTFA, and reagents) were excluded from further analyses. Obtained normalised (using the total ion current (TIC) approach and LOWESS algorithm) results were then exported to Excel for pre-formatting and then used for statistical analyses. Analyses were performed by the Laboratory of Mass Spectrometry, Institute of Bioorganic Chemistry, Polish Academy of Sciences, Poznan, Poland.

Then, a data matrix containing feature name (identified compound name), sample information (four biological replicates per sample), and relative abundance (calculated by peak area) was prepared and submitted to MetaboAnalyst (https://www.metaboanalyst.ca/, accessed on 18 September 2021). After this, the data matrix was performed using three categories of normalisation, including normalisation by median square root data transformation and auto-scaling via the embedded algorithm of the online data analysis software MetaboAnalyst. The differential metabolites were screened by parameters including fold change FC > |2.0|, VIP value > 1, and *p*-value < 0.05. Then, these candidates were marked for pathway identification to explore their biological roles during seed germination.

### 4.5. Statistical Analysis

All data collected for *T. baccata* and *J. communis* plants were analysed separately for the species.

Data describing plant morphology were subjected to an analysis of variance (ANOVA) for height and diameter, as well as for the number of female generative structures (flowers/arils/ovules/cones), with the fertilisation treatment and genotype as fixed effects and individual plants as a random effect. Correlations between the number of female generative structures produced per 1 cm plant height were also analysed by the fertilisation treatment.

We described female reproductive effort by quantitative and qualitative parameters. We used the following quantitative parameters to describe the reproductive effort of female plants: (1) mean number of female strobili produced per 1 cm of main shoot height, (2) mean number of matured arils/cones produced per 1 cm of main shoot height, (3) mean number of matured arils/cones produced per 10 cm of branch length, percentage of developing arils/cones in relation to (4) the number of produced female flowers, and (5) the number of flowers produced, as well as (6) mean number of seeds produced per 1 cm of the main shoot height and (7) seed mass produced per 1 cm of the main shoot height measured at time of seed collection. As quantitative parameters, we used seed mass, seed morphometric parameters, and presence of an embryo as well as N, C and C/N ratio, and metabolite profile. Data describing efficiency of seed production were tested using Student’s *t*-test after data were arcsine transformed or chi-square tests for independent data. Data describing the parameters of seed mass and morphology (seed curved length (SL), seed curved width (SW) and seed projected area) were statistically analysed using ANOVA, with genotype and fertilisation treatment as fixed effects.

In addition, data describing metabolome activity were analysed with ANOVA. Data obtained from the analysis of C/N ratios in seeds were transformed by arcsine and then subsequently analysed with a Student’s *t*-test. Correlations between seed parameters were run using algorithms in R (www.r-project.org, accessed on 16 September 2021) applying the R studio interface and the *ggpairs* function from the GGally package [78]. The line plots and bar plots were generated using the ggplot2 package [79].

Data obtained from GC-MS descriptions of seeds metabolites were also analysed via Principal Component Analysis, and correlation and pattern analyses were done using MetaboAnalyst 2.0, a comprehensive tool suite for metabolomic data analysis (http://metaboanalyst.ca/, accessed on 18 September 2021; [80]), following data log_10_ transformation and scaling manipulations.

All presented data represent the mean ± standard error (1 SE). Means were considered significantly different at *p <* 0.05.

## 5. Conclusions

Although our results come from pot experiments and only from two woody species, they provide some evidence that future changes in atmospheric deposition and future soil composition of N, P and other macroelements could influence seed number, size and quality. By using vegetative-produced plants of species with two different life-histories—*T. baccata* as a model plant of rich environments and *J. communis* as a model of a pioneer species—we showed different patterns of resource allocation in seeds and different impacts of macronutrient deposition in soil on the plants’ reproductive responses. In a rich environment, *T. baccata* and *J. communis* plants produced numerous seeds with higher seed mass; however, fertilised *T. baccata* plants had similar proportions of C and N amounts, C/N ratio, and P and Mg amounts as well as profile of metabolites, whereas fertilised *J. communis* plants had higher proportion of C, N, P and K content, lower C/N ratio, and different profiles for 18 metabolites when compared with their non-fertilised counterparts. We observed that by increasing the investment of time into seed development, *T. baccata* plants in low-nutrient environment could produce the same quality of seeds as fertilised plants. Moreover, we showed that resource availability within the maternal environment is an important factor that affected the reproductive potential of female plants in long-lived woody species. These results suggest that seed size is not a seed-neutral feature, and together with C/N ratio and metabolite profile can be used as marker of seed quality.

## Figures and Tables

**Table 1:** Differential metabolites between *Juniperus communis* L. seeds produced by plants grown in fertilised (F) and non-fertilised (NF) conditions.

Compound	Log2 (FC F/NF) ^1^	*p*-Value	VIP Value ^2^
Asparagine (-dehydrated)	4.54	0.016	1.608
Pyroglutamic acid	4.04	0.016	1.611
L-Aspartic acid	3.61	0.045	1.493
Glucosamine	3.46	0.010	1.643
L-Serine	3.24	0.014	1.623
L-5-Oxoproline	2.89	0.026	1.579
Trans-4-Hydroxy-L-proline	2.70	0.027	1.553
Phenylalanine	2.50	0.027	1.581
N-α-Acetyl lysine	2.03	0.040	1.512
Xylose	−1.03	0.041	1.507
4-Hydroxybenzoic acid	−1.21	0.009	1.646
Sucrose	−1.28	0.033	1.532
D-(−)-Ribose	−1.40	0.041	1.504
4-Hydroxyphenylacetic acid	−1.44	0.009	1.652
Stearic acid	−2.03	0.027	1.555
D-Lyxose	−2.23	0.027	1.554
D-(−)-Arabinose	−2.24	0.027	1.560
Erythrose	−2.95	0.032	1.539

^1^ Fold change of different metabolites between F and NF dry seeds is reported as Log2 (FC F/NF). ^2^ VIP value—weighted sum of squares of the PLS weight, which indicates the importance of the variable to the whole model.

## Data Availability

The dataset analysed during the current study is available from the corresponding author on reasonable request.

## Supplementary Materials

The following supporting information can be downloaded at: https://www.mdpi.com/article/10.3390/ijms23063187/s1.

## References

[1] Hampton J.G., Boelt B., Rolston M.P., Chastain T.G. (2013). Effects of elevated CO_2_ and temperature on seed quality. J. Agric. Sci..

[2] Obeso J.R. (2002). The costs of reproduction in plants. New Phytol..

[3] Saatkamp A., Cochrane A., Commander L., Guja L.K., Jimenez-Alfaro B., Larson J., Nicotra A., Poschlod P., Silveira F.A.O., Cross A.T. (2019). A research agenda for seed-trait functional ecology. New Phytol..

[4] Castro J. (1999). Seed mass versus seedling performance in Scots pine: A maternally dependent trait. New Phytol..

[5] Suárez-Vidal E., Sampedro L., Zas R. (2017). Is the benefit of larger seed provisioning on seedling performance greater under abiotic stress?. Environ. Exp. Bot..

[6] Obeso J.R., Alvarez-Santullano M., Retuerto R. (1998). Sex ratios, size distributions, and sexual dimorphism in the dioecious tree Ilex aquifolium (Aquifoliaceae). Am. J. Bot..

[7] De Frenne P., Blondeel H., Brunet J., Carón M.M., Chabrerie O., Cougnon M., Cousins S.A.O., Decocq G., Diekmann M., Graae B.J. (2018). Atmospheric nitrogen deposition on petals enhances seed quality of the forest herb Anemone nemorosa. Plant Biol..

[8] Naegle E.R., Burton J.W., Carter T.E., Rufty T.W. (2005). Influence of seed nitrogen content on seedling growth and recovery from nitrogen stress. Plant Soil.

[9] del Cacho M., Peñuelas J., Lloret F. (2013). Reproductive output in Mediterranean shrubs under climate change experimentally induced by drought and warming. Perspect. Plant Ecol. Evol. Syst..

[10] Vacek Z., Vacek S., Prokůpková A., Bulušek D., Podrázský V., Hůnová I., Putalová T., Král J. (2020). Long-term effect of climate and air pollution on health status and growth of *Picea abies* (L.) Karst. peaty forests in the Black Triangle region. Dendrobiology.

[11] Dentener F., Drevet J., Lamarque J.F., Bey I., Eickhout B., Fiore A.M., Hauglustaine D., Horowitz L.W., Krol M., Kulshrestha U.C. (2006). Nitrogen and sulfur deposition on regional and global scales: A multimodel evaluation. Glob. Biogeochem. Cycles.

[12] Bobbink R., Hicks K., Galloway J., Spranger T., Alkemade R., Ashmore M., Bustamante M., Cinderby S., Davidson E., Dentener F. (2010). Global assessment of nitrogen deposition effects on terrestrial plant diversity: A synthesis. Ecol. Appl..

[13] Lu C., Tian H. (2017). Global nitrogen and phosphorus fertilizer use for agriculture production in the past half century: Shifted hot spots and nutrient imbalance. Earth. Syst. Sci. Data.

[14] McGroddy M.E., Daufresne T., Hedin L.O. (2004). Scaling of C:N:P stoichiometry in forests worldwide: Implications of terrestrial redfield-type ratios. Ecology.

[15] Meunier C.L., Gundale M.J., Sánchez I.S., Liess A. (2016). Impact of nitrogen deposition on forest and lake food webs in nitrogen-limited environments. Glob. Chang. Biol..

[16] Takemoto B.K., Bytnerowicz A., Fenn M.E. (2001). Current and future effects of ozone and atmospheric nitrogen deposition on California’s mixed conifer forests. For. Ecol. Manag..

[17] Callahan H.S., Del Fierro K., Patterson A.E., Zafar H. (2008). Impacts of elevated nitrogen inputs on oak reproductive and seed ecology. Glob. Chang. Biol..

[18] Marschner H. (1995). Mineral Nutrition of Higher Plants.

[19] Li R., Chen L., Wu Y., Zhang R., Baskin C.C., Baskin J.M., Hu X. (2017). Effects of cultivar and maternal environment on seed quality in Vicia sativa. Front. Plant Sci..

[20] Talhelm A.F., Burton A.J., Pregitzer K.S., Campione M.A. (2013). Chronic nitrogen deposition reduces the abundance of dominant forest understory and groundcover species. For. Ecol. Manag..

[21] Li Y., Yang H., Xia J., Zhang W., Wan S., Li L. (2011). Effects of increased nitrogen deposition and precipitation on seed and seedling production of Potentilla tanacetifolia in a temperate steppe ecosystem. PLoS ONE.

[22] Pers-Kamczyc E., Tyrała-Wierucka Ż., Rabska M., Wrońska-Pilarek D., Kamczyc J. (2020). The higher availability of nutrients increases the production but decreases the quality of pollen grains in *Juniperus communis* L. J. Plant Physiol..

[23] Pers-Kamczyc E., Iszkuło G., Rabska M., Wrońska-Pilarek D., Kamczyc J. (2019). More isn’t always better—The effect of environmental nutritional richness on male reproduction of *Taxus baccata* L. Environ. Exp. Bot..

[24] Pierce S., Bottinelli A., Bassani I., Ceriani R.M., Cerabolini B.E.L. (2014). How well do seed production traits correlate with leaf traits, whole-plant traits and plant ecological strategies?. Plant Ecol..

[25] Garbarino M., Weisberg P.J., Bagnara L., Urbinati C. (2015). Sex-related spatial segregation along environmental gradients in the dioecious conifer, Taxus baccata. For. Ecol. Manag..

[26] Gruwez R., De Frenne P., De Schrijver A., Vangansbeke P., Verheyen K. (2017). Climate warming and atmospheric deposition affect seed viability of common juniper (*Juniperus communis*) via their impact on the nutrient status of the plant. Ecol. Res..

[27] Hultine K.R., Grady K.C., Wood T.E., Shuster S.M., Stella J.C., Whitham T.G. (2016). Climate change perils for dioecious plant species. Nat. Plants.

[28] Petry W.K., Soule J.D., Iler A.M., Chicas-Mosier A., Inouye D.W., Miller T.E.X., Mooney K.A. (2016). Sex-specific responses to climate change in plants alter population sex ratio and performance. Science.

[29] Simancas B., Juvany M., Cotado A., Munné-Bosch S. (2016). Sex-related differences in photoinhibition, photo-oxidative stress and photoprotection in stinging nettle (*Urtica dioica* L.) exposed to drought and nutrient deficiency. J. Photochem. Photobiol. B Biol..

[30] Montesinos D., De Luís M., Verdú M., Raventós J., García-Fayos P. (2006). When, how and how much: Gender-specific resource-use strategies in the dioecious tree *Juniperus thurifera*. Ann. Bot..

[31] Nowak K., Giertych M.J., Pers-Kamczyc E., Thomas P.A., Iszkuło G. (2021). Defence is a priority in female juveniles and adults of *Taxus baccata* L. Forests.

[32] Wu X., Liu J., Meng Q., Fang S., Kang J., Guo Q. (2021). Differences in carbon and nitrogen metabolism between male and female *Populus cathayana* in response to deficient nitrogen. Tree Physiol..

[33] Kumar S., Kumari R., Sharma V. (2014). Genetics of dioecy and causal sex chromosomes in plants. J. Genet..

[34] Cronk Q., Müller N.A. (2020). Default sex and single gene sex determination in dioecious plants. Front. Plant Sci..

[35] Thomas P.A., Polwart A. (2003). *Taxus baccata* L. J. Ecol..

[36] Thomas P.A., El-Barghathi M., Polwart A. (2007). Biological Flora of the British Isles: *Juniperus communis* L. J. Ecol..

[37] Gruwez R., Leroux O., De Frenne P., Tack W., Viane R., Verheyen K. (2013). Critical phases in the seed development of common juniper (*Juniperus communis*). Plant Biol..

[38] Robakowski P., Pers-Kamczyc E., Ratajczak E., Thomas P.A.P.A., Ye Z.-P.P., Rabska M., Iszkuło G. (2018). Photochemistry and antioxidative capacity of female and male *Taxus baccata* L. acclimated to different nutritional environments. Front. Plant Sci..

[39] Nowak K., Giertych M.J., Pers-Kamczyc E., Thomas P.A., Iszkuło G. (2021). Rich but not poor conditions determine sex-specific differences in growth rate of juvenile dioecious plants. J. Plant Res..

[40] Ward L.K. (2007). Lifetime sexual dimorphism in *Juniperus communis* var. communis. Plant Species Biol..

[41] Rabska M., Pers-Kamczyc E., Żytkowiak R., Adamczyk D., Iszkuło G. (2020). Sexual dimorphism in the chemical composition of male and female in the dioecious tree, *Juniperus communis* L., growing under different nutritional conditions. Int. J. Mol. Sci..

[42] Nowak-Dyjeta K., Giertych M.J., Thomas P., Iszkuło G. (2017). Males and females of *Juniperus communis* L. and *Taxus baccata* L. show different seasonal patterns of nitrogen and carbon content in needles. Acta Physiol. Plant..

[43] Huijser P., Schmid M. (2011). The control of developmental phase transitions in plants. Development.

[44] Ohya I., Nanami S., Itoh A. (2017). Dioecious plants are more precocious than cosexual plants: A comparative study of relative sizes at the onset of sexual reproduction in woody species. Ecol. Evol..

[45] Bogdziewicz M., Crone E.E., Steele M.A., Zwolak R. (2017). Effects of nitrogen deposition on reproduction in a masting tree: Benefits of higher seed production are trumped by negative biotic interactions. J. Ecol..

[46] DiFazio S.P., Wilson M.V., Vance N.C. (1998). Factors limiting seed production of Taxus brevifolia (Taxaceae) in western Oregon. Am. J. Bot..

[47] Sanz R., Pulido F. (2015). Pollen limitation and fruit abortion in a declining rare tree, the Eurasian yew (*Taxus baccata* L.): A reproductive cost of ecological marginality. Plant Biosyst. Int. J. Deal. Asp. Plant Biol..

[48] Rabska M., Robakowski P., Ratajczak E., Żytkowiak R., Iszkuło G., Pers-Kamczyc E. (2021). Photochemistry differs between male and female *Juniperus communis* L. independently of nutritional availability. Trees Struct. Funct..

[49] Moles A.T., Westoby M. (2006). Seed size and plant strategy across the whole life cycle. Oikos.

[50] Gómez J.M. (2004). Bigger is not always better: Conflicting selective pressures on seed size in Quercus ilex. Evolution.

[51] Leishman M.R., Wright I.J., Moles A.T., Westoby M. (2009). The evolutionary ecology of seed size. Seeds Ecol. Regen. Plant Communities.

[52] Melzack R.N., Watts D. (1982). Variations in seed weight, germination, and seedling vigour in the Yew (*Taxus baccata* L.) in England. J. Biogeogr..

[53] Jacquemart A.L., Buyens C., Delescaille L.M., Van Rossum F. (2021). Using genetic evaluation to guide conservation of remnant *Juniperus communis* (Cupressaceae) populations. Plant Biol..

[54] Tylkowski T. (2009). Improving seed germination and seedling emergence in the Juniperus communis. Dendrobiology.

[55] Gruwez R., De Frenne P., De Schrijver A., Leroux O., Vangansbeke P., Verheyen K. (2014). Negative effects of temperature and atmospheric depositions on the seed viability of common juniper (*Juniperus communis*). Ann. Bot..

[56] Gaufichon L., Rothstein S.J., Suzuki A. (2016). Asparagine metabolic pathways in arabidopsis. Plant Cell Physiol..

[57] Tian X., Fang Y., Jin Y., Yi Z., Li J., Du A., He K., Huang Y., Zhao H. (2021). Ammonium detoxification mechanism of ammonium-tolerant duckweed (*Landoltia punctata*) revealed by carbon and nitrogen metabolism under ammonium stress. Environ. Pollut..

[58] Santos-Sánchez N.F., Salas-Coronado R., Hernández-Carlos B., Villanueva-Cañongo C. (2019). Shikimic acid pathway in biosynthesis of phenolic compounds. Plant Physiol. Asp. Phenolic Compd..

[59] Kaur M., Tak Y., Bhatia S., Asthir B., Lorenzo J.M., Amarowicz R. (2021). Crosstalk during the carbon–nitrogen cycle that interlinks the biosynthesis, mobilization and accumulation of seed storage reserves. Int. J. Mol. Sci..

[60] Jösson K.I., Tuomi J. (1994). Costs of reproduction in a historical perspective. Trends Ecol. Evol..

[61] Barradas M.C.D., Correia O. (1999). Sexual dimorphism, sex ratio and spatial distribution of male and female shrubs in the dioecious species *Pistacia lentiscus* L. Folia Geobot..

[62] Teitel Z., Pickup M., Field D.L., Barrett S.C.H. (2015). The dynamics of resource allocation and costs of reproduction in a sexually dimorphic, wind-pollinated dioecious plant. Plant Biol..

[63] Queenborough S.A., Burslem D.F.R.P., Garwood N.C., Valencia R. (2007). Determinants of biased sex ratios and inter-sex costs of reproduction in dioecious tropical forest trees. Am. J. Bot..

[64] Wheelwright N.T., Logan B.A. (2004). Previous-year reproduction reduces photosynthetic capacity and slows lifetime growth in females of a neotropical tree. Proc. Natl. Acad. Sci. USA.

[65] Lloyd D.G., Webb C.J. (1977). Secondary sex characters in plants. Bot. Rev..

[66] Rocheleau A.-F.F., Houle G. (2001). Different cost of reproduction for the males and females of the rare dioecious shrub *Corema conradii* (Empetraceae). Am. J. Bot..

[67] Smith C.C., Fretwell S.D., McGinley M.A., Charnov E.L., Smith C.C., Fretwell S.D. (1974). The optimal balance between size and number of offspring. Am. Nat..

[68] McGinley M.A., Charnov E.L. (1988). Multiple resources and the optimal balance between size and number of offspring. Evol. Ecol..

[69] Paul-Victor C., Turnbull L.A. (2009). The effect of growth conditions on the seed size/number trade-off. PLoS ONE.

[70] Montesinos D., Villar-Salvador P., García-Fayos P., Verdú M., Villar-Salvador P., García-Fayos P., Verdú M. (2012). Genders in *Juniperus thurifera* have different functional responses to variations in nutrient availability. New Phytol..

[71] Benham S.E., Houston Durrant T., Caudullo G., de Rigo D. (2016). Taxus baccata in Europe: Distribution, habitat, usage and threats. Eur. Atlas For. Tree Species.

[72] Enescu C.M., Houston Durrant T., Caudullo G., de Rigo D., San-Miguel-Ayanz J., de Rigo D., Caudullo G., Houston Durrant T., Mauri A. (2016). Juniperus communis in Europe: Distribution, habitat, usage and threats. European Atlas of Forest Tree Species.

[73] McCartan S.A., Gosling P.G. (2013). Guidelines for seed collection and stratification of Common Juniper (*Juniperus communis* L.). Tree Plant. Notes.

[74] Załęski A., Gładysz A., Kowalska I. (1991). Ocena nasion przy użyciu promieni X. Pr. Inst. Badaw. Leśnictwa.

[75] (2009). ISO 11885:2007.

[76] Yang X., Feng L., Zhao L., Liu X., Hassani D., Huang D. (2018). Effect of glycine nitrogen on lettuce growth under soilless culture: A metabolomics approach to identify the main changes occurred in plant primary and secondary metabolism. J. Sci. Food Agric..

[77] Wei S., Yang X., Huo G., Ge G., Liu H., Luo L., Hu J., Huang D., Long P. (2020). Distinct metabolome changes during seed germination of lettuce (*Lactuca sativa* L.) in response to thermal stress as revealed by untargeted metabolomics analysis. Int. J. Mol. Sci..

[78] Emerson J.W., Green W.A., Schloerke B., Crowley J., Cook D., Hofmann H., Wickham H. (2012). The Generalized Pairs Plot. J. Comput. Grephical Stat..

[79] Wickham H. (2016). R Package “ggplot2”: Elegant Graphics for Data Analysis.

[80] Xia J., Psychogios N., Young N., Wishart D.S. (2009). MetaboAnalyst: A web server for metabolomic data analysis and interpretation. Nucleic Acids Res..

