# Supplementary material for: Seed Quantity or Quality?—Reproductive Responses of Females of Two Dioecious Woody Species to Long-Term Fertilisation
Source: Int J Mol Sci. 2022 Mar 16;23(6):3187. doi: 10.3390/ijms23063187 (PMC8948795; doi:10.3390/ijms23063187)
Supplement: Supplementary file 1 [file ijms-23-03187-s001.zip › ijms-1576295-supplementary.pdf]

International Journal of
Molecular Sciences

MDPI

Article

Seed Quantity or Quality?—Reproductive Responses of Females of Two Dioecious Woody Species to Long-Term Fertilisation

Emilia Pers-Kamczyc [1,*], Ewa Mąderek [1] and Jacek Kamczyc [2]

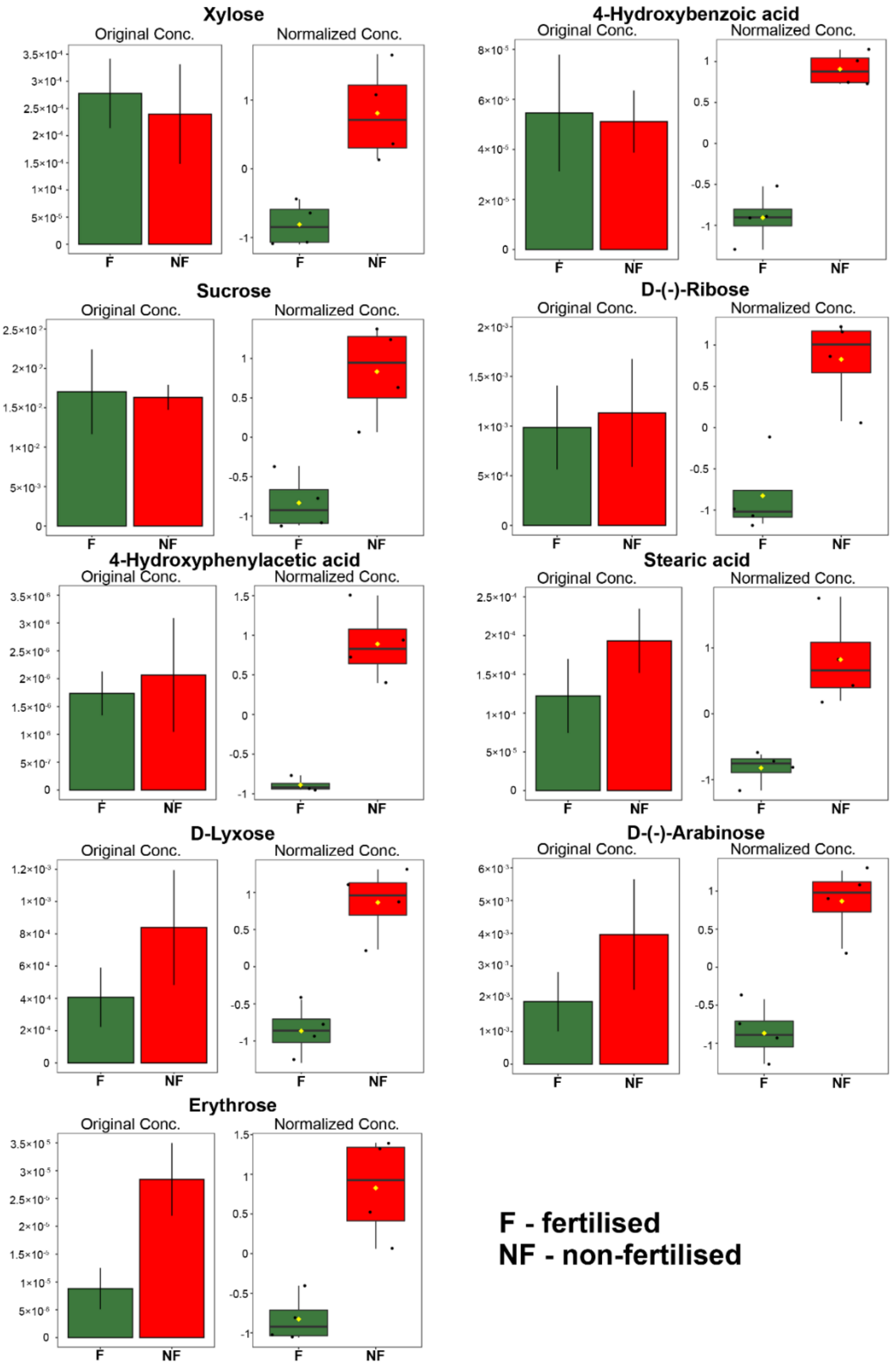

Figure S1. Down-regulated metabolites (original and normalized concentration) of dry seeds collected from *Juniperus communis* L. plants grown in fertilised (F, green) compared to non-fertilised (NF, red) conditions.

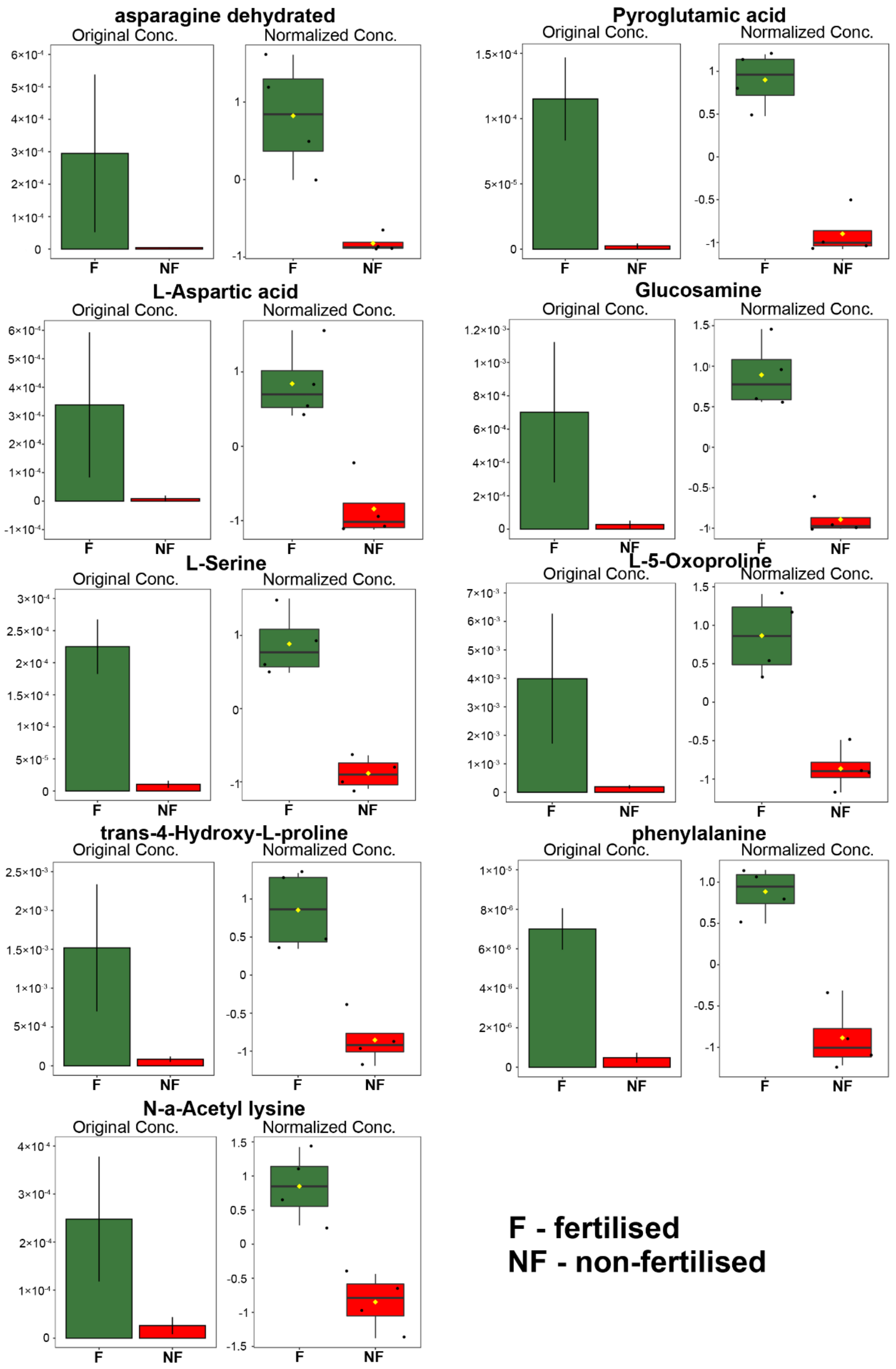

Figure S2. Up-regulated metabolites (original and normalized concentration) of dry seeds collected from *Juniperus communis* L. plants grown in fertilised (F, green) compared to non-fertilised (NF, red) conditions.
